# Amorphous oxide semiconductor for monolithic 3D DRAM: an enabler or passer-by?

**DOI:** 10.1093/nsr/nwad290

**Published:** 2023-11-21

**Authors:** Shujuan Mao, Guilei Wang, Chao Zhao

**Affiliations:** Beijing Superstring Academy of Memory Technology, China; Beijing Superstring Academy of Memory Technology, China; Beijing Superstring Academy of Memory Technology, China

## Abstract

Whether amorphous oxide semiconductor (AOS) is an enabler or pass-by for monolithic 3D DRAM is discussed, with current challenges and future directions proposed in this perspective.

Since dynamic random-access memory (DRAM) was invented in 1968 and put to market in the 1970s, 1 transistor plus 1 capacitor (1T1C) (Fig. 1a) cells have been used to take advantage of their structural simplicity and ease of production. The off-state leakage of the Si-based cell transistor plays a key role in controlling the retention time of the datum, which is saved as the capacitor charges. A cell transistor needs to have a large on-state current and small off-state leakage. Recently, a new amorphous oxide semiconductor (AOS) termed IGZO has attracted worldwide attention because of its properties ideal for DRAM cell transistors, such as high carrier mobility and large energy bandgap (Fig. 1b). Such an amorphous mixture of ZnO, In_2_O_3_, and Ga_2_O_3_ was first reported by Hosono in 2004 [1] and industrially applied in 2013 for manufacturing organic light-emitting diode displays.

Different from Si-based transistors, whose channel must be single crystal, the IGZO transistor has an amorphous channel, which means that it could be easily deposited in any step of the processing sequence. This means that one could stack the cell transistors on peripheral Si logic circuitry, and enable monolithic 3D (M3D) integration of DRAM (Fig. 1c). Such an M3D integration is expected to increase bandwidth, reduce access latency, and achieve a high density of DRAM [2].

Various device structures, such as dual gate [3], channel all around [4], and gate all around [5] have been proposed for IGZO transistors, and excellent performance such as *I*_on_/*I*_off_ >10^11^, with an *I*_off_ <10^−22^ A/μm has been demonstrated [4]. Based on their remarkable leakage control, new cell structures such as two transistors without a capacitor (2T0C) was proposed (Fig. 1a), where the cell capacitor is replaced by a ‘read’ transistor and the datum is saved as the transistor state; 2T0C might lead to a revolutionary solution of multi-bit DRAM [4,6,7] (Fig. 1d).

**Figure 1.**
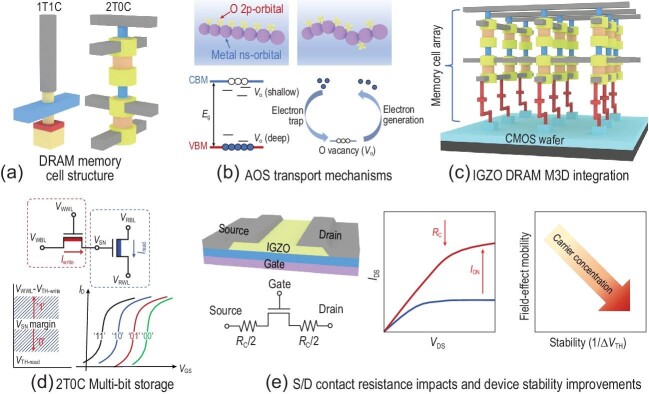
Schematic of critical steps in implementing AOS-based M3D DRAM: deep understanding of (a) DRAM memory cell structure, (b) AOS transport mechanisms, (c) IGZO DRAM M3D integration, (d) 2T0C multi-bit storage and (e) S/D contact resistance impacts and and improvements in device stability.

IGZO DRAM, however, is still in its infancy and faces plenty of challenges. First, the interface resistance at the IGZO/metal contact has to be reduced to an acceptable level. To date, most metals yield Schottky contact with IGZO, instead of ohmic contact, leading to high contact resistances which in turn compromise drive current gain from gate length scaling. Therefore, reducing contact resistances will be a critical factor for IGZO DRAM shrinking (Fig. 1e).

Second, the scalability of an IGZO transistor limits it to stacking integration, which strongly depends on the stability of the bottom transistors during the processing of the top layer. The degradation of an IGZO transistor has been widely observed due to defects in the film or at the interface of the channel/gate dielectric, including intrinsic defects (such as oxygen vacancy (*V_o_*) and interstitial oxygen (*O_i_*)), and extrinsic adsorbates (such as oxygen and water molecules). Hydrogen impurities are an important source of *V_o_* and *O_i_*. How to control these defects will be a determining factor in the future of IGZO DRAM.

Looking into the conduction mechanism of IGZO, the spatial overlap of larger metal *s* orbitals (Fig. 1b), provides the conduction pathway with carrier conduction occurring via transport percolation. The intrinsic defect energy levels relative to the conduction band edges determine the stability. Therefore, there is always a trade-off between mobility and stability. At the moment, it is still unclear whether there is an ‘optimal’ defect density allowing good conductivity with conditions of acceptable stability.

The charm of IGZO DRAM research lies in its wonderful material properties and the possibility of technical revolution, as well as in all the uncertainties that call for the contribution of researchers. Once the processing and device meet the target, brand new DRAM architecture is expected to be able to leverage performance, power, and area optimization. This would be the beginning of a new era of DRAM for applications in emerging fields such as artificial intelligence, big data, and the Internet of Things.
